# Orphan Nuclear Receptor NR4A2 Is Constitutively Expressed in Cartilage and Upregulated in Inflamed Synovium From hTNF-Alpha Transgenic Mice

**DOI:** 10.3389/fphar.2022.835697

**Published:** 2022-04-20

**Authors:** Cullen M. Lilley, Andrea Alarcon, My-Huyen Ngo, Jackeline S. Araujo, Luis Marrero, Kimberlee S. Mix

**Affiliations:** ^1^ Department of Biological Sciences, Loyola University New Orleans, New Orleans, LA, United States; ^2^ Department of Orthopaedic Surgery, Louisiana State University Health Sciences Center, New Orleans, LA, United States

**Keywords:** nuclear receptors, NR4A2 gene, NFkB (RelA), TNF-α, cartilage, synovium, inflammation, rheumatoid arthritis

## Abstract

Orphan nuclear receptor 4A2 (NR4A2/Nurr1) is a constitutively active transcription factor with potential roles in the onset and progression of inflammatory arthropathies. NR4A2 is overexpressed in synovium and cartilage from individuals with rheumatoid arthritis (RA), psoriatic arthritis, and osteoarthritis. This study documents the expression and tissue localization of NR4A2 and upstream regulator nuclear factor kappa B (NF-κB) in the human tumor necrosis factor-alpha (hTNF-α) transgenic mouse model of RA. Since TNF-α is a potent inducer of NR4A2 *in vitro*, we hypothesized that NR4A2 would also be upregulated and active during disease progression in this model. Expression levels of NR4A2, related receptors NR4A1 (Nur77) and 3 (NOR1), and NF-κB_1_ transcripts were quantified by RT-qPCR in hTNF-α and wild-type joints at three stages of disease. The protein distribution of NR4A2 and NF-κB subunit RelA (p65) was analyzed by quantitative immunohistochemistry. Global gene expression of 88 RA-related genes was also screened and compared between groups. Consistent with previous reports on the hTNF-α model, transgenic mice exhibited significant weight loss and severely swollen paws by 19 weeks of age compared to age-matched wild-type controls. NR4A1-3 and NF-κB_1_ were constitutively expressed at disease onset and in healthy joints. NF-κB_1_ transcript levels increased 2-fold in hTNF-α paws with established disease (12 weeks), followed by a 2-fold increase in NR4A2 at the late disease stage (19 weeks). NR4A2 and RelA proteins were overexpressed in inflamed synovium prior to symptoms of arthritis, suggesting that gene expression changes documented in whole paws were largely driven by elevated expression in diseased synovium. Broader screening of RA-related genes by RT-qPCR identified several differentially expressed genes in hTNF-α joints including those encoding inflammatory cytokines and chemokines, matrix-degrading enzymes and inhibitors, cell surface receptors, intracellular signaling proteins and transcription factors. Consensus binding sites for NR4A receptors and NF-κB_1_ were enriched in the promoters of differentially expressed genes suggesting central roles for these transcription factors in this model. This study is the first comprehensive analysis of NR4A2 in an animal model of RA and validates the hTNF-α model for testing of small molecules and genetic strategies targeting this transcription factor.

## Introduction

Rheumatoid arthritis (RA) is a chronic inflammatory arthropathy with an estimated global population prevalence of 0.46% (Almutairi et al., 2021). RA is characterized by synovial membrane hyperplasia, leukocyte infiltration, and irreversible cartilage and bone destruction in multiple joints. Biological therapies targeting the inflammatory cytokine tumor necrosis factor-alpha (TNF-α) serve as robust treatment options for attenuating chronic inflammation in RA (Mitoma et al., 2018; Kerschbaumer et al., 2020). However, these agents are expensive and 30%–40% of RA patients have inadequate clinical responses (Rubbert-Roth et al., 2019). Furthermore, blocking TNF-α results in broad-spectrum immunosuppression that increases the risk of serious infections and some types of cancer (Sartori et al., 2019; Sepriano et al., 2020; Li et al., 2021). Elucidating molecular pathways downstream of TNF-α may lead to the development of more selective drugs capable of attenuating joint damage without compromising essential immune functions.

The orphan nuclear receptor 4A2 (NR4A2/Nurr1) may be a promising therapeutic target downstream of TNF-α and nuclear factor kappa B (NF-κB) signaling pathways. Τhis transcription factor is a member of the NR4A family of receptors along with NR4A1 (Nur77) and NR4A3 (NOR1). The NR4A receptors share a high degree of homology and may have functional redundancy in some cellular contexts (Crean and Murphy, 2021). In contrast to other nuclear receptors, the NR4A receptors are presumed to be constitutively active, ligand-independent factors regulated at the level of expression and post-translational modification (Zhao and Bruemmer, 2010). In response to inflammation, NF-κB and cyclic adenosine monophosphate response element binding protein (CREB) bind directly to the NR4A2 promoter and rapidly induce its expression in chondrocytes, synoviocytes, endothelial cells, and immune cells (McEvoy et al., 2002; Ralph et al., 2005; Pei et al., 2006; Mix et al., 2007). NR4A2 is also highly expressed in inflamed synovial tissues from individuals with RA and psoriatic arthritis as well as in cartilage from individuals with osteoarthritis (OA) (Murphy et al., 2001; McEvoy et al., 2002; Ralph et al., 2005; Mix et al., 2007; Aherne et al., 2009; Ralph et al., 2010; Mix et al., 2012). Over-expression of NR4A2 in synovial fibroblasts enhances proliferation, anchorage-independent growth, and invasion, suggesting critical roles for this receptor in synovial hyperplasia (Mix et al., 2012)*.* At the transcriptional level, NR4A2 regulates expression of the chemokine interleukin 8 (IL-8), cartilage-degrading matrix metalloproteinases-1 and 13 (MMP-1, 13), and the immunomodulatory peptide hormone prolactin (Davies et al., 2005; Mix et al., 2007; Aherne et al., 2009; Mix et al., 2012; McCoy et al., 2015). While analyses of human joint tissues and cells have yielded important insight into NR4A2-dependent mechanisms, a comprehensive analysis of receptor mRNA expression levels and protein distribution has not been performed in an animal model of arthritis.

This study provides a detailed analysis of gene expression levels and joint tissue distribution patterns of the NR4A receptors and NF-κB in a transgenic mouse model of RA driven by chronic expression of the human TNF-α cytokine (hTNF-α, Taconic model 1006). Transgenic models expressing hTNF-α have demonstrated great utility for pre-clinical validation of therapies and insight into RA mechanisms (Douni et al., 2004; Zwerina et al., 2004; Delavallée et al., 2009; Binder et al., 2013; Li et al., 2016; Karagianni et al., 2019; Ubah et al., 2019). The hTNF-α transgenic mice studied here exhibit spontaneous and progressive inflammation leading to severe polyarthritis by 20 weeks of age. Since TNF-α is a potent inducer of NF-κB and NR4A2 in human joint cells, we hypothesized that these transcription factors would also be upregulated and active during disease progression in the hTNF-α model. To test this, we quantified NR4A1-3 and NF-κB mRNA levels by RT-qPCR and measured protein distribution by immunohistochemistry in joints from hTNF-α mice at different disease stages. In addition, a broader screen of RA-related genes was conducted and potential NR4A and NF-κB target genes were identified through promoter analyses. Our results provide the first spatiotemporal map of NR4A2 distribution in an animal model of RA and validate the hTNF-α model for testing of small molecules and genetic therapies targeting this transcription factor.

## Methods and Materials

### Animals

Male hTNF-α transgenic and C57BL6/N wild-type mice were obtained from Taconic Biosciences (Model 1006; Hudson, NY, United States). The hTNF-α model was generated using a 2.8 kb transgene containing the human TNF-α gene with the full-length promoter and coding region. The endogenous 3′ untranslated region (UTR) of the human TNF-α gene was replaced with the human beta-globin 3′ UTR which served to stabilize the transcript (Keffer et al., 1991). The transgenic line was produced by pronuclear microinjection of B6SLJ (F2) hybrid zygotes and mice were backcrossed for over 20 generations onto the C57BL6/N genetic background. For this study, transgenic and wild-type littermates were maintained at Taconic Biosciences until 8 (*n* = 5), 12 (*n* = 4), and 19 weeks of age (*n* = 4), representing early, established, and late stages of RA (Taconic Biosciences, 2022). Mice were group-housed in a barrier facility with a 12-h light cycle and access to food and water ad libitum. At the end of the study, body mass was measured and clinical scores were assessed by Taconic Biosciences using a 24-point scoring system as follows: 20 digits scored as 0 (normal) or 0.2 (one or more swollen joints) for a maximum total digit score of 4, each paw scored as 0 (normal), 1 (noticeably swollen) or 2 (severely swollen) for a maximum total paw score of 8, each wrist scored as 0 (normal), 1 (noticeably swollen), or 2 (severely swollen) for a maximum total wrist score of 4 and each ankle scored as 0 (normal), 2 (noticeably swollen), or 4 (severely swollen) for a maximum total ankle score of 8 (Taconic Biosciences, 2022). Immediately after euthanasia, paws were dissected proximal to the ankle/wrist joints and transferred to RNAlater (left paws) and 10% neutral buffered formalin (right paws) for RT-qPCR and histology, respectively. Protocols were approved by the Institutional Animal Care and Use Committees at Taconic Biosciences and Loyola University New Orleans.

### Immunohistochemistry and Imaging

Hind paws (*n* = 13 per genotype) were fixed in neutral-buffered formalin followed by decalcification with formic acid-based Decalcifier I (Leica), paraffin processed and embedded sagittally for microtomy. Serial 5 µm-thick sections were cut and mounted on Trubond 380 slides (Electron Microscopy Sciences). Slides were then heated for 45 min at 59°C, deparaffinized, re-hydrated, and formaldehyde cross-links were dissociated by submerging slides in pressure-heated citrate buffer (pH 6.0). Endogenous peroxidases were removed by treatment with 3% H_2_O_2_ in methanol and non-specific binding was reduced by incubation in Protein Block (Abcam). Sections were incubated overnight at 4°C with either rabbit polyclonal anti-NR4A2 (Novus NB110-40415), rabbit monoclonal anti-RelA (Abcam ab32536), rabbit IgG isotype control (Abcam 172730) or antibody diluent only. After washing, sections were incubated for 30 min in anti-rabbit Ig (H + L) secondary conjugated to horseradish peroxidase (Vector ImPRESS). The reaction product of Diaminobenzidine and H_2_O_2_ was used as substrate to visualize the tagged epitopes. Slides were counterstained with hematoxylin, mounted, and images collected with an Olympus BX51 microscope equipped with a digital camera driven by CellSens software (Olympus). Semi-quantitative scoring of RelA and NR4A2-positive cells in synovium and cartilage from at least two 400× fields per section was conducted by two blinded observers. Representative images from hTNF-α and wild-type joints at 400× magnification are shown.

### RNA Extraction and RT-qPCR

Contralateral paws were dissected proximal to the ankle/wrist joints and the skin was removed. Tissue was coarsely minced with a scalpel and homogenized with a TissueTearor (Thomas Scientific, Swedesboro, NJ, United States). Total RNA was extracted using RNeasy fibrous tissue mini columns (Qiagen, Germantown, MD, United States), eluted into RNase-free water and quantified with a NanoDrop spectrophotometer (Thermo Fisher Scientific). RNA was reverse transcribed into complementary DNA (cDNA) using the iSCRIPT reverse transcriptase master mix (Bio-Rad, Hercules, CA, United States). Twenty microliter reactions were prepared with 1 µg of total RNA from each paw (50 ng/µl cDNA). Control reactions were prepared with the negative control master mix provided with the kit. Reactions were incubated in a Bio-Rad C1000 thermocycler for 5 min at 25°C, 30 min at 42°C, and 5 min at 85°C. Quantitative PCR was performed using iTaq Universal SYBR Green SuperMix (Bio-Rad, Hercules, CA, United States) and validated primers spanning exon-intron junctions in the mouse TATA-Box Binding Protein (TBP), NR4A1, NR4A2, NR4A3, and NF-κB_1_ genes (Bio-Rad, Hercules, CA, United States). Twenty microliter reactions were prepared in triplicate with 50 ng of cDNA from each sample and incubated in a CFX96 Real-Time PCR Detection System (Bio-Rad, Hercules, CA, United States) for 30 s at 95°C, 5 s at 95°C, 30 s at 60°C (40 cycles), followed by melting curve analysis from 65°C–95°C using CFX Manager Software (Bio-Rad, Hercules, CA, United States). Relative gene expression was calculated by the 2^−ΔΔCt^ method (Livak and Schmittgen, 2001) with target gene levels normalized to TBP.

### RT-qPCR Panels and Promoter Analysis

Pre-designed PrimePCR panels were used to analyze 88 mouse genes associated with RA and reference genes (Rheumatoid Arthritis Tier 1 M96, Bio-Rad, Hercules, CA, United States). Pooled cDNA from transgenic or wild-type paws (*n* = 4/group) at early or late time points was mixed with iTaq Universal SYBR Green SuperMix (Bio-Rad, Hercules, CA, United States) and added to each well of the PrimePCR reaction plates containing lyophilized primers. Reactions were incubated for 30 s at 95°C, 5 s at 95°C, 30 s at 60°C (40 cycles), followed by melting curve analysis from 65°C–95°C. Target gene expression was normalized to TBP and Glyceraldehyde 3-phosphate dehydrogenase (GAPDH) using the gene study analysis in the CFX Manager Software. Control assays included on each PrimePCR plate confirmed RNA integrity and the absence of contaminating genomic DNA. Thresholds for induced and repressed genes in hTNF-α mice were set at a >2-fold increase or a >0.5-fold decrease, respectively. Genes were classified as stable if fold-change values were between these thresholds. LASAGNA (Length-Aware Site Alignment Guided by Nucleotide Association)-Search, an integrated web tool for predicting transcription factor binding sites with the LASAGNA algorithm, was used to analyze gene promoters in all expressed genes (Lee and Huang, 2013). Promoter regions of mouse genes (−1,000 bp to 0) were scanned for NR4A2 (AAGGTCAC) and NF-κB_1_ (GGGGATTCCCC) binding sites using matrix derived JASPAR CORE models of consensus vertebrate binding sites, with a *p*-value threshold of <0.001.

### Statistical Analysis

**FIGURE 1:**
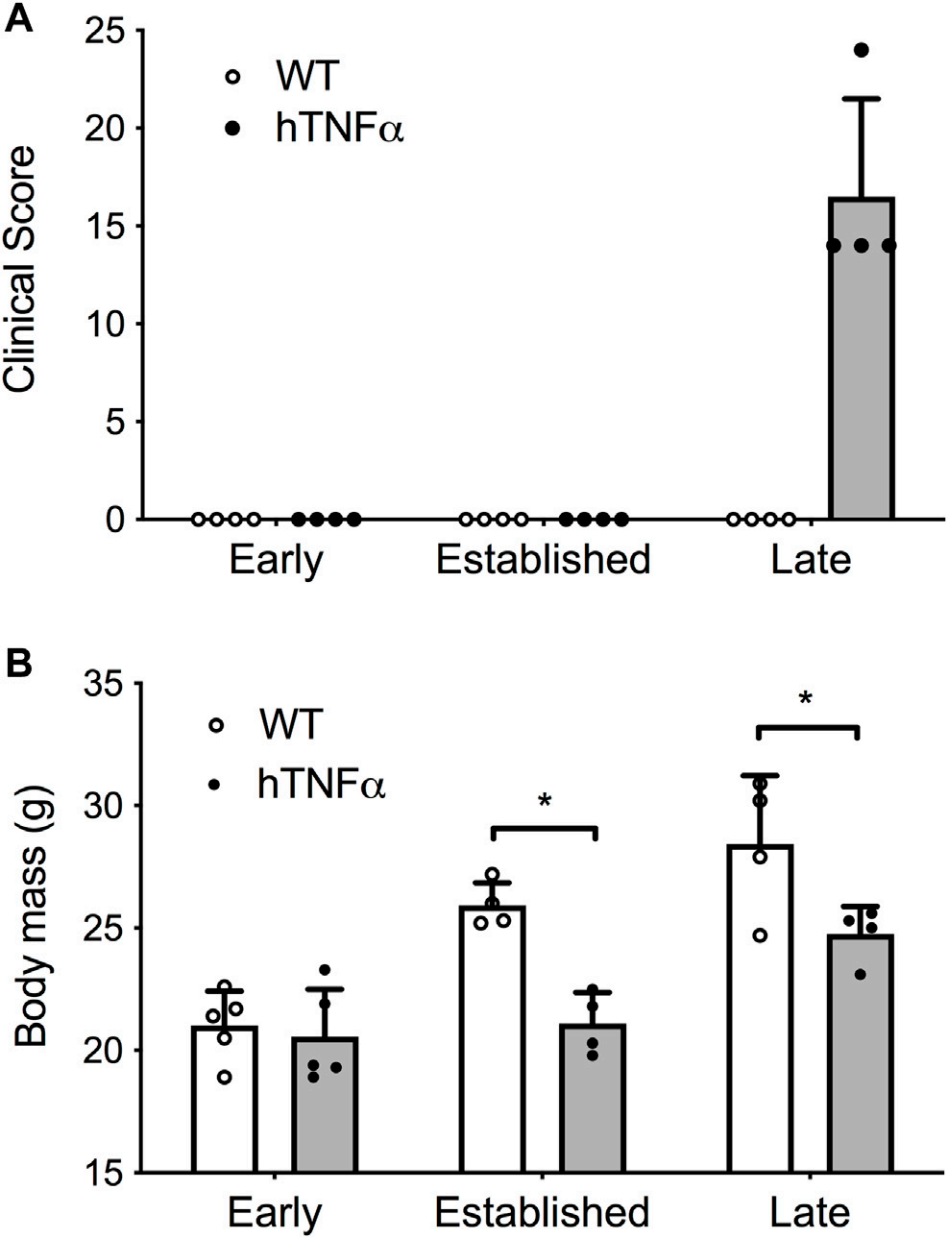
Progression of arthritis in hTNF-α transgenic mice. Wild-type and hTNF-α mice were studied at three stages of disease: early (8 weeks), established (12 weeks), and late (19 weeks). **(A)** Mean clinical score based on total paw swelling ±standard deviation. **(B)** Mean body mass (g) ± standard deviation. Unpaired *t*-test, *p < 0.05.

**FIGURE 2:**
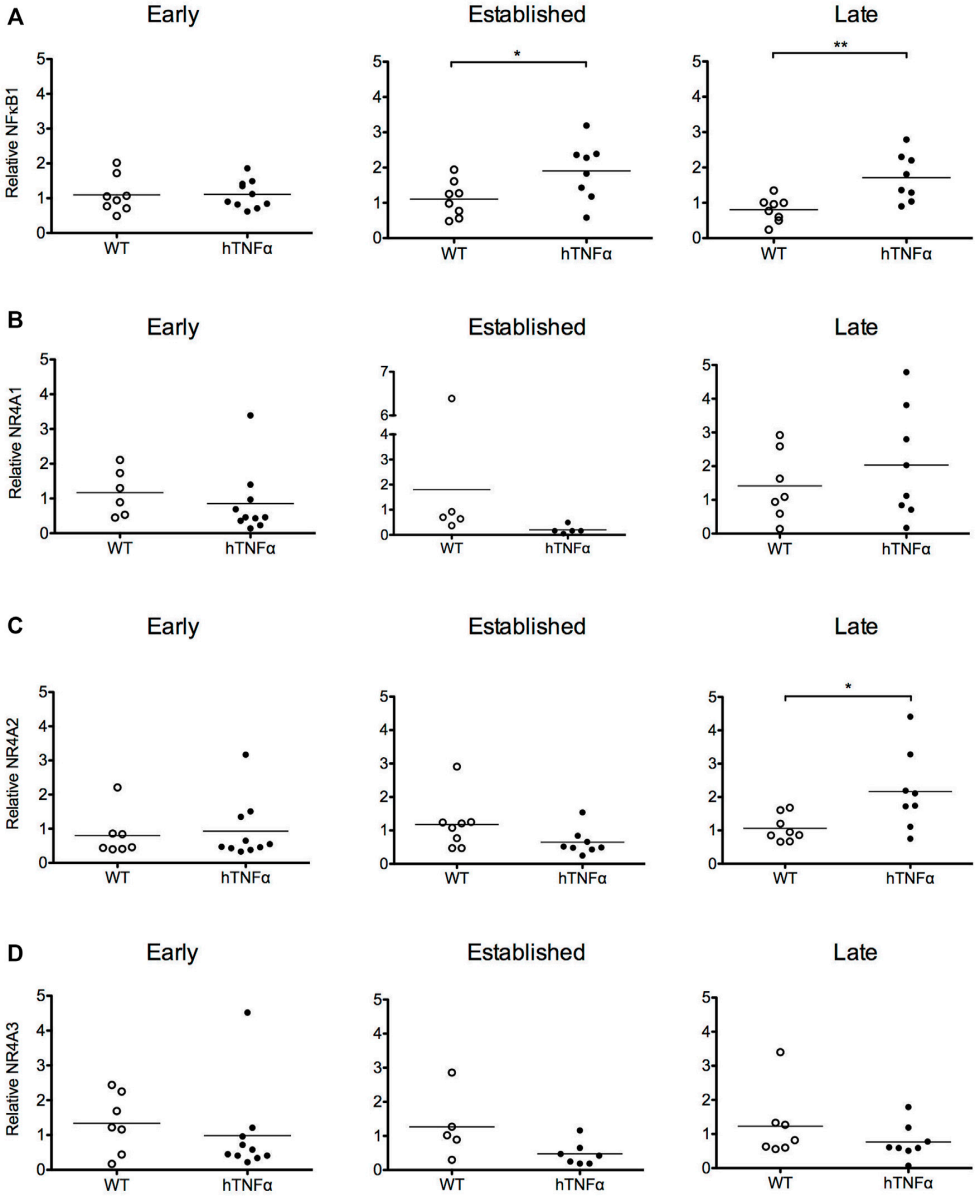
Gene expression analysis of target transcription factors. RNA was extracted from the paws of wild-type and hTNF-α transgenic mice at early, established, and late timepoints. Relative levels of NF-κB_1_ **(A)**, NR4A1 **(B)**, NR4A2 **(C)**, NR4A3 mRNA **(D)** were measured by RT-qPCR and normalized to TBP. Horizontal bars represent mean expression levels for each group. Mann Whitney test, *p < 0.05, **p < 0.005.

**FIGURE 3:**
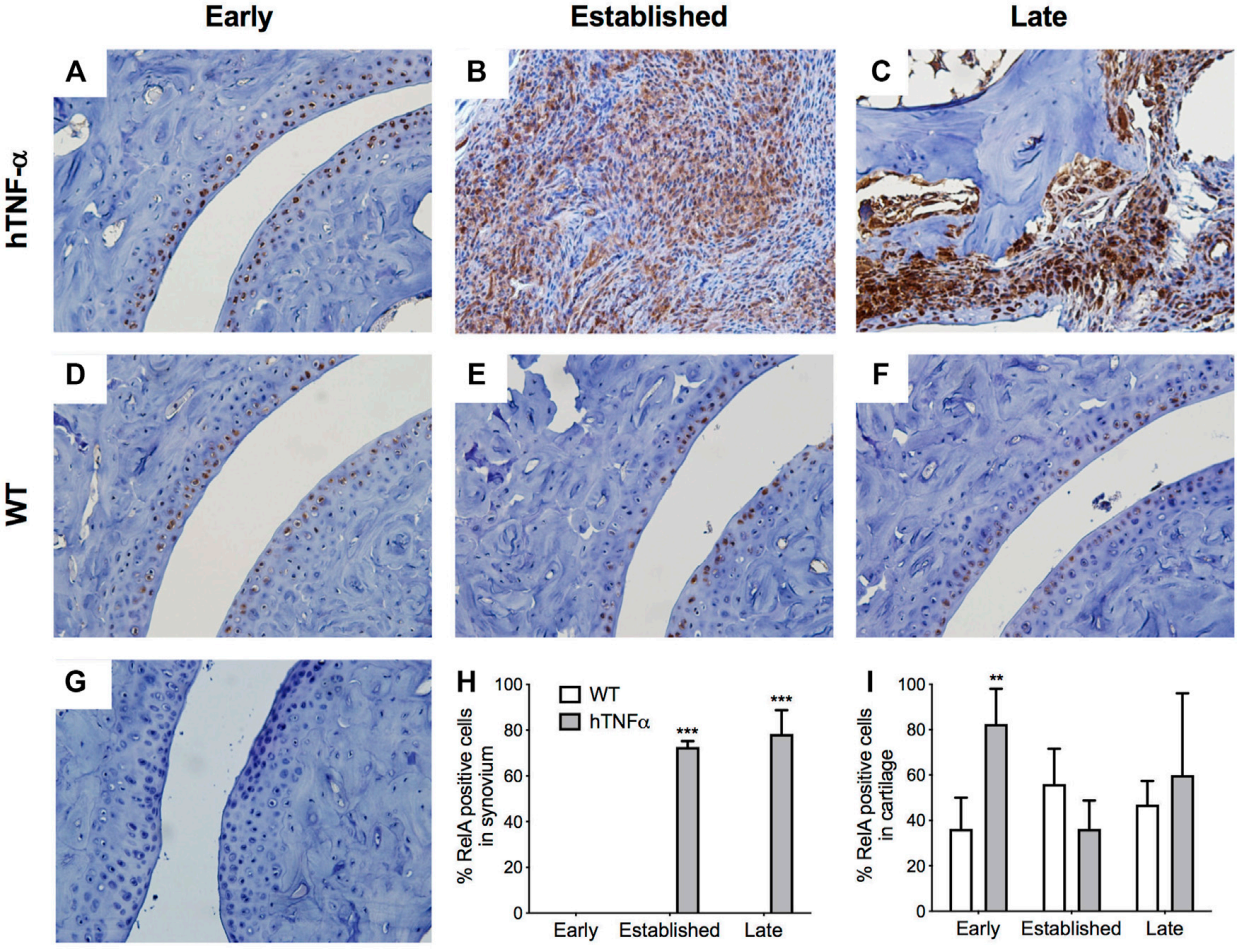
Localization of RelA in cartilage and synovium. RelA was detected in paws of wild-type and hTNF-α mice at early **(A,D)**, established **(B,E)**, and late **(C,F)** stages of disease by immunohistochemistry. Isotype control was applied to a transgenic section from the late stage of disease **(G)**. Representative images at 400× magnification are shown. Scoring of RelA positive cells in synovium **(H)** and cartilage **(I)**. Unpaired *t*-test, **p < 0.005, ***p < 0.0005.

**FIGURE 4:**
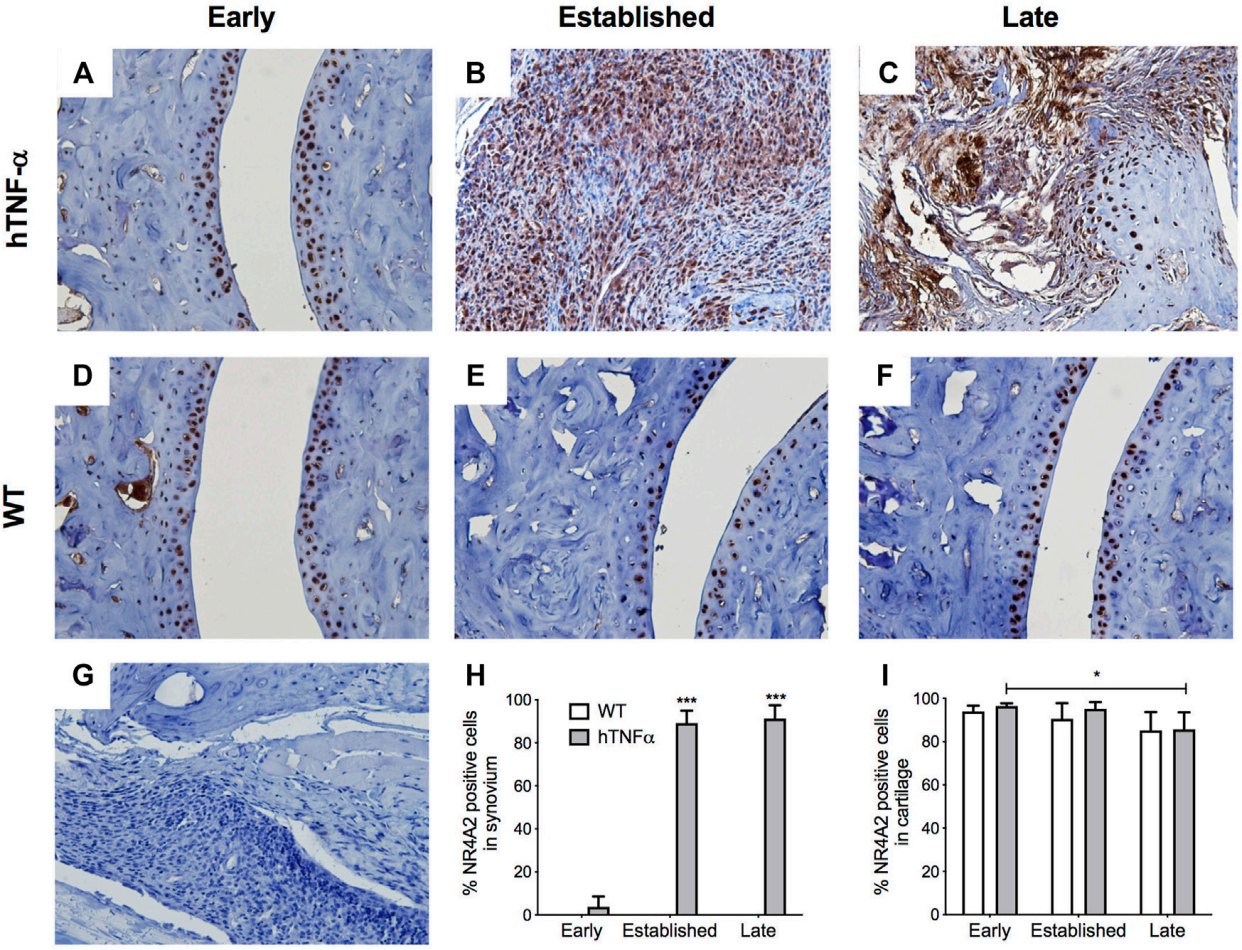
Localization of NR4A2 in cartilage and synovium. NR4A2 protein was detected in paws of wild-type and hTNF-α mice at early **(A,D)**, established **(B,E)**, and late **(C,F)** stages of disease by immunohistochemistry. Isotype control was applied to a transgenic section from the late stage of disease **(G)**. Representative images at 400× magnification are shown. Scoring of NR4A2 positive cells in synovium **(H)** and cartilage **(I)**. Unpaired *t*-test, *p < 0.05, ***p < 0.0005.

GraphPad Prism software (version 9.3.1) was used to generate graphs and compare gene and protein expression data between groups of age-matched transgenic and wild-type mice. Normality was confirmed with the D’Agostino-Pearson test and the unpaired *t*-test was applied to data in Figures 1, 3, 4 with the threshold of statistical significance at *p* < 0.05. The Mann Whitney test was used to analyze RT-qPCR data with deviations from normality in Figure 2 with a threshold of statistical significance at *p* < 0.05.

## Results

Symptoms and physical signs of arthritis were monitored in hTNF-α transgenic mice at early (8 weeks), established (12 weeks), and late (19 weeks) disease stages. Transgenic mice appeared healthy at 8 weeks of age but demonstrated impaired mobility and reduced digit splaying by 12 weeks. Severe polyarthritis was evident in all transgenic mice by 19 weeks and elevated clinical scores reflected severely swollen digits, paws, and ankle joints (Figure 1A). Consistent with previous studies in this model (Taconic Biosciences, 2022), synovial hyperplasia, leukocyte infiltration, cartilage destruction, fibrosis, and subchondral bone erosion were observed in digit and ankle joints (data not shown). Furthermore, hTNF-α mice demonstrated a significant decrease in body mass at 12 and 19 weeks (Figure 1B, *p* < 0.05), consistent with RA-induced cachexia. In contrast, wild-type mice remained healthy and demonstrated a steady increase in body mass.

To investigate transcriptional pathways activated by hTNF-α signaling in this model, NF-κB and orphan nuclear receptor NR4A1-3 mRNA levels were measured in whole paws from transgenic and wild-type mice by RT-qPCR. NF-κB_1_ mRNA was expressed at similar levels between transgenic and wild-type groups at 8 weeks and increased 2-fold at established and late disease stages (Figure 2A, *p* < 0.05). All three NR4A receptors were detected without differential expression at 8 and 12 weeks (Figures 2B–D). At the late disease stage, NR4A2 was selectively upregulated 2-fold (Figure 2C, *p* < 0.05), while NR4A1 and 3 remained equivalent to wild-type levels. These gene expression results reflect the net expression patterns of NF-κB_1_ and the NR4A receptors in whole paws composed of multiple tissues and cell types.

To expand on these findings and measure the histological distribution of NF-κB and NR4A2 proteins, sections of contralateral paws from hTNF-α and wild-type mice were assayed by immunohistochemistry. RelA was detected in chondrocytes from wild-type and hTNF-α joints (Figures 3A,D,E,F). At the early stage of disease, 80% of hTNF-α chondrocytes in the resting zone of the articular surfaces stained positive for RelA (Figure 3I, *p* < 0.005). However, at the established and late disease stages, RelA positivity in cartilage decreased to wild-type levels without significant differences between groups Figures 3E,F,I. Synovial hyperplasia was observed with concurrent increases in RelA signal in synoviocytes (Figures 3B,H, >70%, *p* < 0.005) and cells in synovio-entheseal compartments (Figure 3C). Isotype controls confirmed the absence of background staining on paw sections (Figure 3G).

NR4A2 was detected in a similar distribution pattern in both cartilage and synovium and ubiquitously expressed in superficial chondrocytes from hTNF-α and wild-type joints at the early timepoint (Figures 4A,D,I). A 10% decrease in NR4A2 positive chondrocytes was observed in both groups at late stage, but statistical significance was limited to the hTNF-α groups (Figure 4E,F,I, *p* < 0.05). Synovial hyperplasia coincided with increases in NR4A2 in the synovium (>80%) and abundant expression in synoviocytes at the sites of membrane insertion to the cartilage (Figures 4B,C,H, *p* < 0.0005). Isotype controls confirmed the absence of background staining on paw sections (Figure 4G). Taken together, elevated RelA and NR4A2 protein levels in synoviocytes suggest that gene expression changes noted in whole paws (Figure 2) are largely driven by disease changes in the synovium.

**FIGURE 5:**
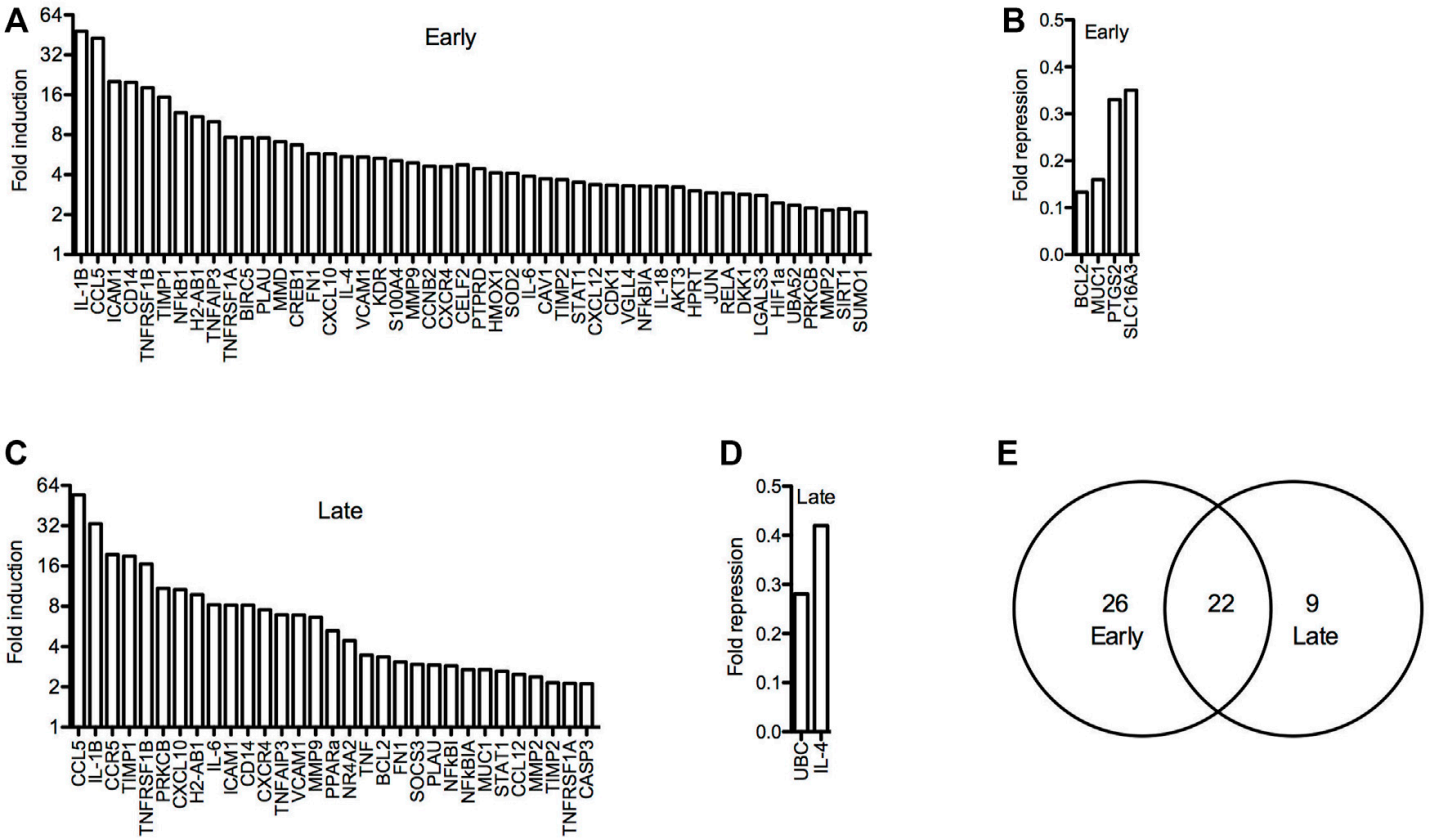
Global gene expression profiles in hTNF-α joints. RT-qPCR panels were used to measure the expression of 88 genes associated with RA in wild-type and hTNF-α paws at early and late stages of disease. **(A)** Genes induced by greater than 2-fold at early stage. **(B)** Genes repressed by greater than 0.5-fold at early stage. **(C)** Genes induced by greater than 2-fold at late stage. **(D)** Genes repressed by greater than 0.5-fold at late stage. **(E)** Comparison of induced genes at early and late stages.

To further investigate arthritis-related molecular mechanisms in the hTNF-α model, global gene expression patterns were examined in paws using RT-qPCR panels specific for 88 genes implicated in RA. A set of 48 genes was upregulated at the early stage of disease relative to wild-type controls, of which 22 remained elevated at the late stage along with nine additional induced genes (Figures 5A,C,E). Upregulated genes included inflammatory cytokines and chemokines (IL-1β, IL-6, IL-1, IL-4, CCL5, CXCL10, CXCL12), matrix degrading enzymes and inhibitors (MMP2, MMP9, TIMP1, TIMP2), cell surface receptors (ICAM1, VCAM1, CD14, CCR5, CXCR4, KDR), intracellular signaling proteins (AKT3, SOCS3, PRKCB, S100A4) and transcription factors (JUN, HIF1-alpha, STAT1, PPAR-alpha, CREB1). Endogenous mouse TNF-α was elevated by 3.5-fold (Figure 5C), consistent with autoregulation of this cytokine in response to chronic hTNF-α signaling. Validating RT-qPCR results presented earlier, NR4A2 (+4.4-fold) and NF-κB transcripts (NFKB1 +11.8-fold, RELA +2.9-fold) were also upregulated in hTNF-α paws (Figures 5A,C). Notably, four genes were repressed at the early stage (BCL2, MUC1, PTGS2, SLC16A3) and two were repressed at the late stage (UBC, IL-4) (Figures 5B,D). Differential expression was not observed for the remaining genes detected on the panels (early = 28, late = 51, Supplementary Table S1).

Next, the promoters of all genes detected in the panel experiment were scanned for putative binding sites for NF-κB_1_ (GGGGATTCCCC) and NR4A receptors (AAGGTCAC) using matrix derived JASPAR CORE models of consensus vertebrate binding sites (Lee and Huang, 2013). Consensus binding sites for these factors were enriched in the promoters of differentially expressed genes relative to the stably expressed groups (Table 1). Over half of the early induced promoters contained at least one NR4A or NF-κB_1_ binding site and 38% of the promoters contained sites for both factors. Furthermore, NR4A consensus binding sites were predicted in 8 of the 10 most highly induced promoters (CCL5, ICAM1, CD14, TNFRSF1B, NFκB_1_, H2-AB1, TNFAIP3, TNFRSF1A) (Supplementary Table S2). These trends were evident in differentially expressed genes from early and late stages of disease, suggesting that NF-κB and NR4A receptors regulate transcriptional pathways central to this model.

**TABLE 1:** Consensus binding sites in promoters of differentially and stably expressed genes.

	Early stage			Late stage		
	Induced	Repressed	Stable	Induced	Repressed	Stable
Number of expressed genes	48	4	28	30	2	51
Promoters with NR4A sites	30 (63%)	1 (25%)	10 (36%)	19 (63%)	2 (100%)	22 (43%)
Promoters with NF-κB_1_ sites	28 (58%)	3 (75%)	10 (36%)	16 (53%)	2 (100%)	25 (47%)
Promoters with both sites	18 (38%)	0 (0%)	3 (10%)	12 (40%)	2 (100%)	8 (16%)

## Discussion

This study provides the first comprehensive analysis of the NR4A receptors and NF-κB in a transgenic mouse model of RA driven by the human TNF-α cytokine. Since TNF-α is a potent inducer of NF-κB and NR4A receptors in human joint cells, we hypothesized that these transcription factors would also be expressed in arthritic joints *in vivo*. NF-κB_1_ and NR4A2 mRNA transcripts were upregulated in whole paws from hTNF-α mice, while NR4A1 and NR4A3 were not differentially expressed (Figure 2). Consistent with transcriptional induction of NR4A2 by NF-κB in human joint cells (McEvoy et al., 2002; Ralph et al., 2005; Mix et al., 2007), we documented an increase in NF-κB_1_ mRNA prior to NR4A2 upregulation at the late disease stage. Furthermore, we detected potent increases in RelA and NR4A2 proteins in inflamed synovium by immunohistochemistry (Figures 3, 4), suggesting that gene expression patterns in whole paws are largely driven by changes in the synovium. In contrast, NR4A2 was constitutively expressed in the resting zone of cartilage but decreased at the late stage of disease. Since NR4A2 is a constitutively active transcription factor tightly regulated at the level of expression, the detection of NR4A2 protein in articular surfaces and synovium suggests this receptor is transcriptionally active in resident chondrocytes and synoviocytes.

Our results in the hTNF-α model are consistent with tissue-specific activities for the NR4A receptors in joints. In human synovial fibroblasts, NR4A2 exacerbates inflammation and tissue degradation by upregulating IL-8, MMP-13, prolactin, CRH, and CRH-receptor1 (Murphy et al., 2001; Davies et al., 2005; Ralph et al., 2007; Aherne et al., 2009; Mix et al., 2012; McCoy et al., 2015). In human chondrocytes, NR4A2 antagonizes MMP-1, 3, and 9 gene expression and contributes to chondroprotection (Mix et al., 2007). However, depletion of NR4A1-3 in human chondrocytes antagonizes histamine-dependent regulation of RANKL expression, providing evidence for differential modulation of genes involved in cartilage degradation (Marzaioli et al., 2012). In addition, studies in rat chondrocytes support a protective function for NR4A1 through suppression of COX-2, iNOS, and MMP expression (Xiong et al., 2020), while other studies suggest that NR4A3 has opposing effects (Ma et al., 2020).

By screening a broader panel of genes implicated in RA, we generated evidence of increased expression of various inflammatory cytokines and chemokines, matrix degrading enzymes and inhibitors, cell surface receptors, intracellular signaling proteins and transcription factors that support the hTNF-α mouse as a model of RA (Figure 5). Of these, IL-1β and CCL5 were the most potently induced genes, consistent with the known activation of these promoters in response to increased TNF-α signaling (Turner et al., 1989; Lee et al., 2000). Additionally, NR4A2, NF-κB_1_, and RelA were also upregulated concurrent with increases in endogenous murine TNF-α. This screen focused on a select group of genes involved in RA and as such our results do not reflect the full spectrum of aberrant gene expression changes that may be occurring within hTNF-α joints.

Promoter analysis of differentially expressed genes supports central roles for NF-κB and NR4A transcription factors in the hTNF-α model. Consensus binding sites for NF-κB_1_ were predicted in the promoters of 58% of the induced genes in contrast to only 36% of the stable genes (Table 1). Several of these differentially expressed genes have been previously recognized as transcriptional targets of NF-κB. NR4A monomeric binding sites known as Nurr binding response elements (NBRE: AAACCGTA) were also enriched in the promoters of differentially expressed genes. Among the genes containing these promoter elements (Supplementary Table S2), NR4A receptors have been reported to regulate BCL-2, IL-6, CXCL-12, CXCR-4, and MMP-9 in various cellular contexts (Johnson et al., 2011, Bonta et al., 2006, Mix et al., 2007, Duren et al., 2016). NR4A receptors can also modulate gene expression through heterodimeric binding with retinoid X receptors (RXR) and interactions with NF-κB and erythroblast transformation specific (ETS) transcription factors (Mix et al., 2007; Aherne et al., 2009; Saijo et al., 2009; Duren et al., 2016; McEvoy et al., 2017).

Our findings are supported by recent gene expression studies highlighting the importance of NF-κB and NR4A receptors in the pathophysiology of arthritis. Integrative transcriptome analysis of a distinct hTNF-α transgenic model with rapid onset of symptomatic arthropathy (Tg197, Keffer et al., 1991) ranked NF-κB as the most important transcription factor in this model (Karagianni et al., 2019). Interestingly, the NR4A receptors exhibited disparate expression patterns in limbs from the Tg197 model; NR4A1 and 3 were downregulated and NR4A2 was not significantly altered. The hTNF-α model used here expresses significantly lower levels of hTNF-α and exhibits a gradual onset of symptoms that more closely models chronic RA (Hayward et al., 2007). Another study of synovial gene expression profiles in OA identified NR4A2 as one of the top 10 transcription factors linked to differentially expressed genes from multiple microarray datasets (Fei et al., 2016). However, NR4A2 was categorized as a downregulated gene in these OA synovial datasets, in contrast to its upregulation in OA cartilage (Mix et al., 2007), RA synovium (Murphy et al., 2001; McEvoy et al., 2002; Ralph et al., 2005; Aherne et al., 2009; Ralph et al., 2010), and hTNF-α joints in this study.

NR4A expression and pharmacological targeting have been investigated in other mouse models of arthritis, providing broader insight into the therapeutic potential of these receptors*.* In the K/BxN serum-induced model of RA (Christianson et al., 2012), NR4A1-3 mRNA levels were elevated in inflamed ankles and NR4A2 expression was potently suppressed by dexamethasone and intra-articular injections of salmon calcitonin and hyaluronic acid (Ryan et al., 2013). In CD4^+^ T-cells isolated from DBA/1 mice with collagen-induced arthritis (CIA), NR4A1-3 mRNA levels were reduced relative to naïve controls and the NR4A agonist cytosporone B improved clinical scores *in vivo* (Saini et al., 2019). NR4A2 protein was detected in inflamed synovium and cartilage of ankle joints from the TNF^-delta-ARE^ model of chronic inflammation (Kontonyiannis et al., 1999; Smyth et al., 2019) and also in joints from the antigen-induced arthritis model in a pilot study (Everett et al., 2015). Further investigation of the NR4A receptors in animal models of arthritis will provide greater insight into the mechanisms linking these transcription factors to inflammation and cartilage degradation *in vivo.*

Recent advances in synthetic NR4A ligands and endogenous receptor modulators have provided new strategies for targeting the NR4A receptors *in vitro* and *in vivo.* The ligand-binding domain of NR4A2 was once thought to be incompatible with the binding of endogenous ligands (Wang et al., 2003), however structural studies have revealed that unsaturated fatty acids can bind to the canonical NR4A ligand-binding pocket and transactivate the NR4A2 receptor (de Vera et al., 2016; de Vera et al., 2019). Probing interactions between unsaturated fatty acids and NR4A receptors may lead to the development of new synthetic agents for selective receptor targeting. The purine anti-metabolite 6-mercaptopurine activates the NR4A receptors *in vitro,* suggesting some of the therapeutic actions of this widely used chemotherapeutic agent may also be mediated through the NR4A receptors (Ordentlich et al., 2003; Wansa et al., 2003; Wansa and Muscat, 2005). Furthermore, multiple structurally-diverse agents have been identified that can regulate NR4A expression and transcriptional activity as selective agonists or antagonists (Safe et al., 2016; Munoz-Tello et al., 2020). Most relevant to the current study, the synthetic NR4A2 agonist 1,1-bis(3′-indolyl)-1-(p-chlorophenyl) methane (C-DIM12) blocked TNF-α induction of adhesion molecules and NF-κB regulated genes in primary murine synovial fibroblasts (Afzali et al., 2018), suggesting that this agent may be a candidate for *in vivo* testing in arthritis models.

Our study has limitations but presents opportunities for in-depth studies of the impact of NR4A receptors on structural and symptomatic arthritis *in vivo.* This study was restricted to male hTNF-α mice since they exhibit earlier symptoms and a higher degree of arthritis severity (Hayward et al., 2007). Female mice should be included in future studies to address mechanisms of sexual dimorphism in the model. Microdissection of joint tissues would provide greater insight into the distribution of transcription factors and their potential target genes within hTNF-α joints. Furthermore, determining the cell-type-specific expression patterns of the NR4A receptors within the hTNF-α synovium would refine pharmacotherapeutic targeting strategies.

In conclusion our results provide the first spatiotemporal map of NR4A2 distribution in an animal model of arthritis and validate the hTNF-α model for future testing of synthetic ligands and genetic strategies targeting this transcription factor *in vivo*.

## Data Availability

The original contributions presented in the study are included in the article/Supplementary Materials, further inquiries can be directed to the corresponding author.
